# An Active Building Programme at Leicester

**Published:** 1921-07-02

**Authors:** 


### SCHEME TO RAISE £100,000.

### An Active Building Programme at Leicester.

The extension of Leicester Royal Infirmary by the addition of a new wing of 100 beds has involved the provision of additional accommodation for nurses, and on Thursday, June 23, upwards of 700 guests assembled at the opening by Lady Hazlerigg of the sixty-six additional new bedrooms to the nurses' home. The cost of both these additions amounts to £87,000, towards which £10,000 has been received from the British Red Cross Society, £10,000 from the Leicestershire Prisoners of War Fund, and £22,500 from the trustees of the late Mr. T. G. Langham. And at the opening ceremony the Chairman, Mr. T. Fielding Johnson, announced a donation of £10,000 in memory of the late Mr. T. Fielding Johnson (an announcement which covered a personal gift from the Chairman and Mrs. Fielding Johnson). Mr. Johnson said that more than half of this total is for building purposes, and that was a sufficient justification for going forward with the extensions.

### Furniture Made on the Premises.

Lady Hazlerigg, in a delightful speech declaring the new bedrooms open, said that in the present day, when one heard so much about cutting down expenditure, it was quite cheering to find an institution not only able to carry on but to increase its work. She contrasted the work of to-day with that of fifty years ago, when her father, Mr. Buxton, was Chairman and Treasurer of the London Hospital. Speeches followed from Sir Thomas Cope, Bart., and the Rev. F. Lansdown, who proposed thanks to Sir Arthur and Lady Hazlerigg, and from Mrs. Banton and Miss Irwin, J.P., to the Chairman and Mrs. Fielding Johnson. The guests then proceeded to the nurses' home to inspect the new bedrooms. Each room is fitted with polished wooden-frame bedstead and spring mattress, rug, chest of drawers, washstand with marble top, corner dress cupboard, bookcase, writing-table, and radiator—the furniture being made by the staff of the institution in the works department under the care of Mr. Deacon. Adequate bathroom and other accommodation is provided. The floors are of doloment.

### The Chairman's Generosity.

We understand that the casual manner in which the Chairman announced his munificent gift of £10,000 led many people to the conclusion that the £10,000 was one of the bequests of the late Mr. Fielding Johnson. This is not so. The money is the gift of the Chairman himself in memory of his father. The substantial nature of the gift and the unostentatious manner in which it was announced are, writes one who knows him well, characteristic of the Chairman, who dispenses his generosity in an unobstrusive way and whose large-heartedness is not fully realised.

### The 150th Anniversary Gift Scheme.

At an earlier stage of the meeting the House Governor and Secretary submitted a scheme for raising £100,000 as a 150th anniversary gift, and spoke of the progress made in the past twenty years. His suggestion is to invite contributions of units of 12s. 6d. (150 pence representing a penny for each year's service) and to ask the city to raise approximately 120,000 units and the county 48,000 units—being roughly the proportions due for each, taking into consideration the numbers of patients sent. The rateable value of the city has been selected as a basis for the number of units for which individual ratings should be asked to be responsible, while in the county areas parishes of 200 population should be asked to raise fifty units and other parishes in proportion at the rate of one unit for every four of the population. He hoped the scheme would appeal to every section of the community. In order to work this scheme Mr. Harry Johnson has been relieved of much of the administrative work of the hospital, and will forthwith introduce his proposal to representative committees in the city and county.

